# Supplementary material for: Netcombin: An algorithm for constructing optimal phylogenetic network from rooted triplets
Source: PLoS One. 2020 Sep 18;15(9):e0227842. doi: 10.1371/journal.pone.0227842 (PMC7500971; doi:10.1371/journal.pone.0227842)
Supplement: S4 File — (ZIP) [file pone.0227842.s004.zip › SequenceData-Triplet/sequenceData-10-20-30-0-Triplet/length 200, numseq 30, rate 10^-7/8 seq.pdf]

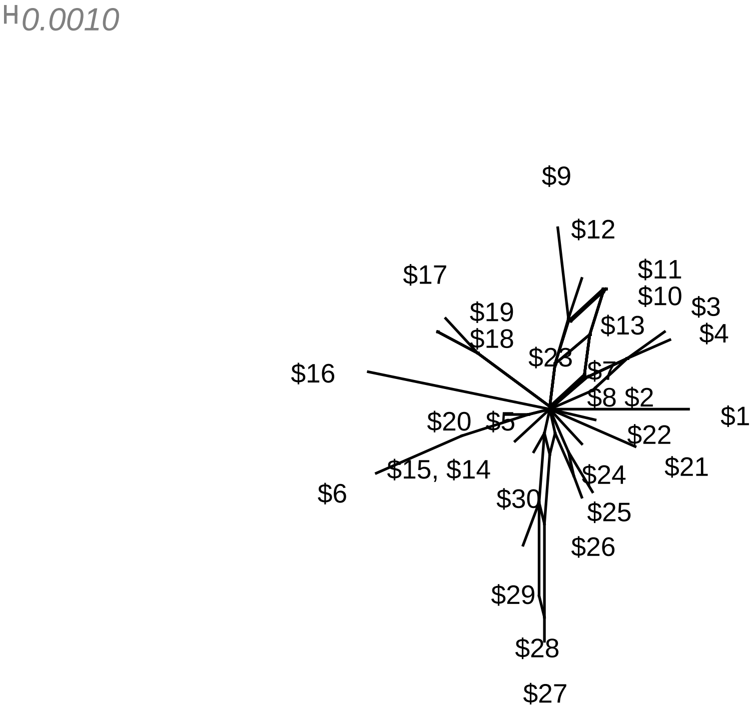

0.0010
$9
$12
$11
$10
$3
$4
$17
$19
$18
$13
$23
$16
$7
$8 $2
$1
$20
$5
$22
$15, $14
$21
$24
$6
$30
$25
$26
$29
$28
$27
